# The Window of Implantation Is Closed by Estrogen via Insulin-Like Growth Factor 1 Pathway

**Published:** 2017

**Authors:** Ryosuke Kobayashi, Jumpei Terakawa, Tsutomu Omatsu, Yupadee Hengjan, Tetsuya Mizutani, Yasushige Ohmori, Eiichi Hondo

**Affiliations:** 1- Laboratory of Animal Morphology, Division of Biofunctional Development, Graduate School of Bioagricultural Sciences, Nagoya University, Nagoya, Japan; 2- Division of Transgenic Animal Science, Advanced Science Research Center, Kanazawa University, Kanazawa, Japan; 3- Research and Education Center for Prevention of Global Infectious Diseases of Animals, Tokyo University of Agriculture and Technology, Tokyo, Japan

**Keywords:** Embryo implantation, Estrogen, Insulin-like growth factor 1, Mouse

## Abstract

**Background:**

The opening and closing of the implantation window is important for successful pregnancy in eutherians. The recent study demonstrated that the window of uterine receptivity was prepared by the sole action of progesterone in mice, but the mechanism to close the window remained to be elucidated.

**Methods:**

The pregnant mice were ovariectomized on the evening on the third day of pregnancy with a single injection of medroxyprogesterone acetate to induce delayed implantation (DI). Several treatments were applied to DI mice. The uterine receptivity after treatment was assessed by examining cell proliferation in the uterine luminal epithelium (LE). The gene expressions in the endometrium were investigated by RNA-seq. The p<0.05 was considered significant.

**Results:**

Cell proliferation in the LE ceased only when the window of implantation was open. Estrogen (E2) stimulated cell proliferation in the LE rendered the uterus refractory. The high throughput gene expression analysis by RNA-Seq showed that the insulin-like growth factor 1 (IGF1) pathway was the candidate to close the implantation window under E2. *In vivo* administration of IGF1 to delayed implantation mice resulted in proliferation in the LE cells.

**Conclusion:**

This study demonstrated that the window of uterine receptivity was closed by E2, which was mediated by the IGF1 pathway.

## Introduction

Embryo implantation initiates placentation and comprises apposition, adhesion, and invasion of the trophoblast in humans and rodents(1). These events occur only for a limited period during early pregnancy, which are known as “the window of implantation” and are regulated by progesterone (P4) and estrogen (E2) (1). In humans, the endometrium can receive embryos only between the seventh and tenth days after ovulation (1). Most cases of spontaneous and/or recurrent miscarriage in humans occur at the time of implantation and are owing to a poor control of the window caused, for instance, by abnormal feto-maternal interactions and/or a refractory uterus (2–4). However, the mechanism for controlling the transition between the receptive and refractory (non-receptive) phases of the endometrium is still poorly understood.

The sensitivity of the mouse uterus for implantation is classified as receptive, neutral, or refractory (5). The uterus becomes receptive to embryos (opening of the window) at the fourth day of pregnancy (D4, the plug≡D1) and refractory (closing of the window) at D5 (5). The neutral phase can be induced experimentally in the delayed-implantation (DI) model, which has been useful for controlling the effects of steroid hormones on implantation (5). A recent study has shown that the first step of the implantation process, the embryo apposition, is initiated by the repression of the effect of E2 on the luminal epithelium (LE) via P4 pathways in mice (6). P4 induces the expression of heart and neural crest derivatives expressed transcript 2 (HAND2) in the sub-epithelial stroma before embryo implantation (D4) (6). HAND2 is regulated by the progesterone receptors (PRs) in the stroma, and probably through the Indian hedgehog (IHH) pathway involving the PRs in the LE (6–9). HAND2 inhibits multiple members of the fibroblast growth factor (FGF) family and eventually suppresses LE cell proliferation with downregulation of E2-regulated genes, such as mucin 1 (Muc 1) (6). The embryo cannot connect to the LE in Hand2-deficient mice (no apposition), in which the LE cells do not stop proliferating (6). That is, the endometrium is prepared to receive embryos, because LE cell proliferation terminates with suppression of the E2 pathway through P4-HAND2 signaling.

The transient increase of nidatory E2 at D4 is also essential for the reception of embryos into the endometrium in mice (10). This E2 induces the temporal expression of leukemia inhibitory factor (LIF), supposed to be mainly expressed in the uterine glands (11). LIF, which substitutes for nidatory E2, potentiates adhesion of the embryo to the LE (11–13). E2 stimulation at D4 induces the receptive phase of the uterus through the LIF pathway. It has been suggested that the uterus spontaneously becomes refractory as implantation progresses (14). Since endometrial receptivity is achieved through suppression of the E2 effect by P4, it is presumed that the uterus became refractory to embryos from D5 onward by resurgence of the E2 effect. Here, in this study, an attempt was made to demonstrate that the transition of the uterus from the receptive phase to the refractory phase (closure of the implantation window) is achieved by E2 action via the insulin-like growth factor 1 (IGF1) signaling, independent of the LIF pathway.

## Methods

### Animals:

All experimental procedures were performed with Avertin anesthesia (Sigma-Aldrich; St. Louis, MO, USA). The ethics committee at Nagoya University approved these all animal experiments (approval number: 2010042803). C57BL/6 mice (Kyudo; Saga, Japan) and ICR mice (Japan SLC; Shizuoka, Japan), aged 6–10 weeks, were used in the experiments. The mice were housed at 23 °*C*±3 °*C* under a controlled light-dark cycle (12 *hr* light, 12 *hr* dark) and were fed ad libitum. The presence of a vaginal plug after mating was designated as the first day of pregnancy (D1). Pseudopregnant female mice were obtained by mating with a vasectomized male.

### Purification of recombinant proteins:

Recombinant LIF, FGF2 and FGF9 (rLIF, rFGF2 and rFGF9, respectively) were prepared as described in a previous report (12). The expression vectors were constructed using pET-46 (Merck, Darmstadt, Germany) with the corresponding proteins described below: LIF, 179 amino acid residues: PLPITPVN ATCAIRHPCHGNLMNQIKNQLAQLNGSANALFISYYTAQGEPFPNNVEKLCAPNMTDFPSFHGNGTEKTKLVELYRMVAYLSASLTNITRDQKVLNPTAVSLQVKLNATIDVMRGLLSNVLCRLCNKYRVGHVDVPPVPDHSDKEAFQRKKLGCQLLGTYKQVISVLAQAF;

FGF2, 145 amino acid residues: PALPEDGGA AFPPGHFKDPKRLYCKNGGFFLRIHPDGRVDGVREKSDPHVKLQLQAEERGVVSIKGVCANRYLAMKEDGRLLASKCVTEECFFFERLESNNYNTYRSRKYSSWYVALKRTGQYKLGSKTGPGQKAILFLPMSAKS;

FGF9, 205 amino acid residues: VGSYFGVQD AVPFGNVPVLPVDSPVLLSDHLGQSEAGGLPRGPAVTDLDHLKGILRRRQLYCRTGFHLEIFPNGTIQGTRKDHSRFGILEFISIAVGLVSIRGVDSGLYLGMNEKGELYGSEKLTQECVFREQFEENWYNTYSSNLYKHVDTGRRYYVALNKDGTPREGTRTKRHQKFTHFLPRPVDPDKVPELYKDILSQS.

Expression vectors were transformed into Escherichia coli BL21 (DE3) cells, and production of the His-tagged recombinant proteins was induced with isopropyl -d-1-thiogalactopyranoside (Wako, Osaka, Japan) for 6 *hr* at room temperature (RT). The proteins were purified using HisTrap FF (GE Healthcare, Little Chalfont, UK), followed by reversed-phase chromatography (trifluoroacctic acid/acetonitrile) with the Proteonavi apparatus (Shiseido, Tokyo, Japan).

### Induction of cell proliferation:

Pseudopregnant mice were ovariectomized in the evening of D3, and siliconized medroxyprogesterone acetate (Depo Provera, 1.0 *mg/mouse*; Pfizer, Inc.; NY, USA) was subcutaneously injected to induce delayed implantation. Three days later, the following treatments were performed either to the mice; 3 *ng* or 20 *ng* of 17β-estradiol (Sigma-Aldrich) by subcutaneous injection, 25 *μg* of rLIF protein, or 200 *μ*g of rFGF2 or rFGF9 protein by intraperitoneal injection, recombinant IGF1 protein (Somazon; 100 *μg/mouse* in every 6 *hr* for 24 *hr*; Orphan Pacific, Tokyo, Japan) by subcutaneous injection, or vehicle (corn oil or phosphate buffered saline). The uteri were collected 24 *hr* after first injection in order to analyze cell proliferation.

### Detection of cell proliferation:

**Figure 1.**
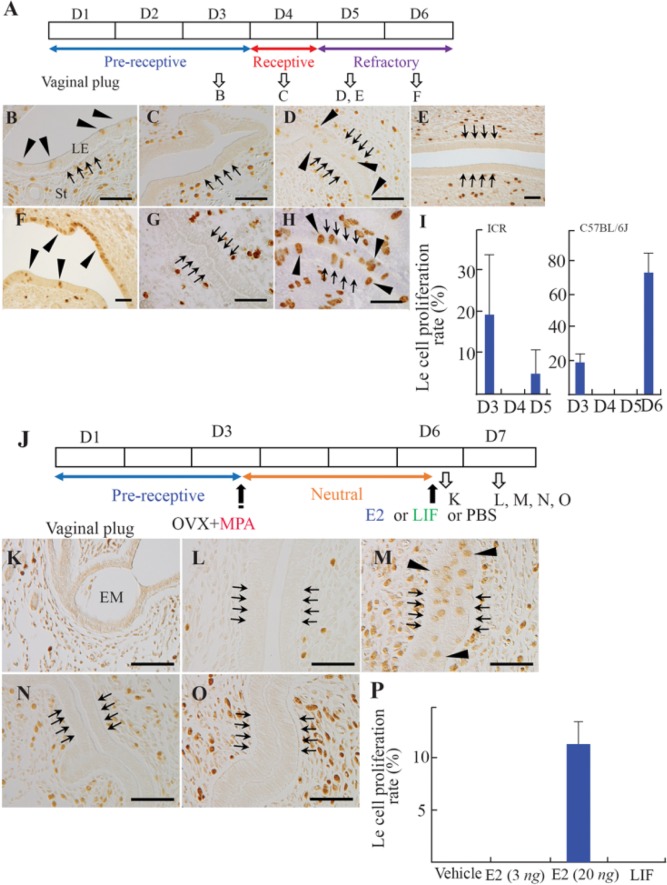
Cell proliferation in the luminal epithelium (LE). (A) The scheme of normal pregnancy. Normal pregnancy on day 3 (D3, B), D4 (C), and D5 (D) in ICR mice, and D5 (E) and D6 (F) in C57BL/6 mice. Pseudopregnant uteri on day 5 (pD5, G) and pD6 (H). (I) The cell proliferation rate during normal pregnancy. (J) Preparation of delayed implantation (DI) model mice. (K) Embryo (Em) apposing to the LE in DI mouse. The uteri of DI mouse injected with vehicle (L), 20 ng of estrogen (E2) (M), 25 *μg* leukemia inhibitory factor (LIF) (N), or 3 *ng* E2 (O). (P) The cell proliferation rate after treatment. St, stroma. Scale bar, *50 μm*

**Figure 5.**
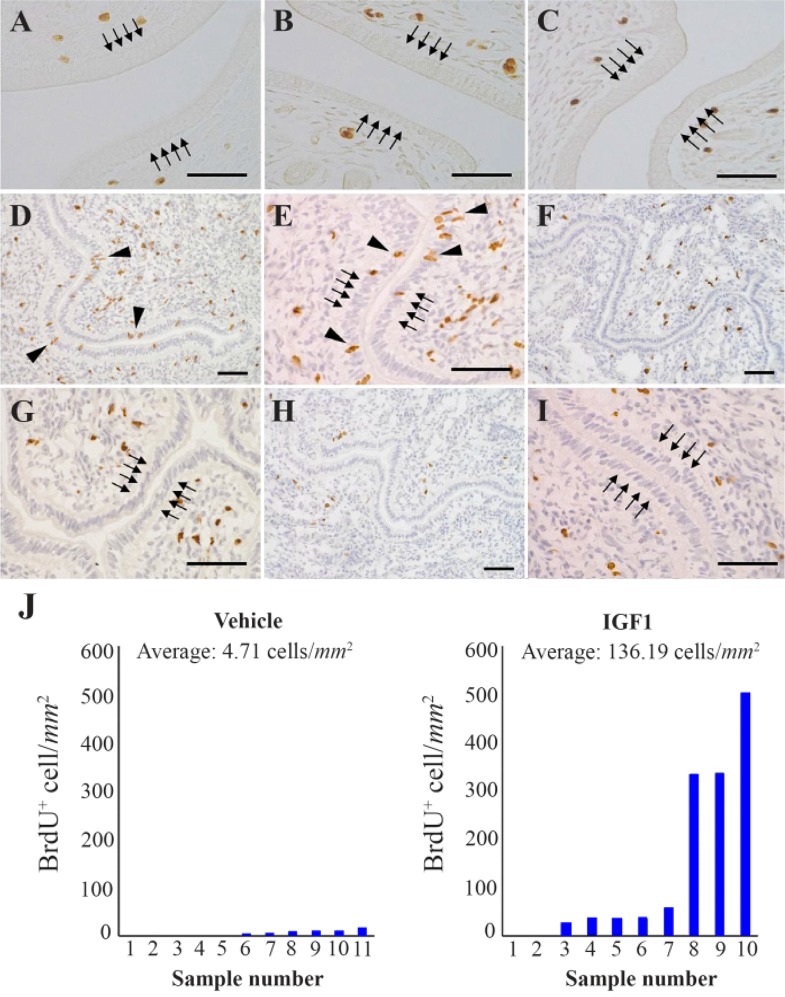
Cell proliferation in the luminal epithelium (LE) followed by treatment with recombinant protein to delayed implantation (DI) mice. The uteri of DI mice intraperitoneally injected with fibroblast growth factor 2 (FGF2, A), FGF9 (B), vehicle (C), or subcutaneously injected with insulin-like growth factor 1 (IGF1), (D, E, F, G and H) or vehicle (H, I). (J) Proliferating cell per unit area of the LE after injection with IGF1. Scale bar, 50 *μm*

An intraperitoneal injection of BrdU (50 *mg/kg*, Sigma-Aldrich) was administered 4.5 *hr* before the uterus was collected. Uteri were fixed in 4% paraformaldehyde and embedded in paraffin, or were embedded in O.C.T. compound (Sakura Finetek, Tokyo, Japan) without fixation and frozen at –80 °*C*. For paraffin- embedded samples, the sections (4 *μm*) were deparaffinized, autoclaved (121 °*C*, 5 *min*), and incubated with 0.3% H_2_O_2_ in methanol for 30 *min* at room temperature (RT); this was followed by incubation with 2N HCl for 30 *min* and with 0.1% trypsin for 20 *min* at 37 °*C*. For frozen samples, the sections (4 *μm*) were fixated by 4% paraformaldehyde for 30 *min*, then incubated with 0.3% H_2_O_2_ in methanol for 30 *min* at RT and incubated with 2N HCl for 60 *min* at 37 °*C*. Monoclonal anti-BrdU antibodies (paraffin-embedded samples; 1: 500, frozen samples; 1:200, Sigma-Aldrich) were used with the Histofine Mouse Stain Kit (Nichirei; Tokyo, Japan). Sections were visualized with 3,3′-diamino benzidine (DAB; Dako; Glostrup, Denmark). Some sections were counterstained with hematoxylin. The ratio of cell proliferation was calculated as the number of BrdU positive LE cells/the total LE cells in figure 1I and 1P. Each experiment was performed at least 3 times. In mice injected with IGF1 (Figure 5), the ratio of cell proliferation was defined as the number of BrdU positive LE cells per unit area of the LE region. The area of the LE region was measured using Image J (National Institutes of Health, Maryland, USA). Five crossing sections of the uterus were randomly selected (at least 100 *μm* interval by uterine longitudinal sectioning), and analyzed for cell proliferation.

### Embryo transfer:

Recipient pseudopregnant mice were ovariectomized in the evening of D3, and siliconized medroxyprogesterone acetate (2.0 *mg/mouse*) was subcutaneously injected. Three days later (D7), 4–5 embryos that were collected from the uteri of normal pregnant females in the morning (0930–1000 h) of D4 were transferred to uteri of pseudopregnant mice. E2 (20 *ng*) or LIF (25 *μg*) treatment was administered 24 *hr* before embryo transfer (D6). A single injection of LIF (25 *μg*) was given 12 *hr* after embryo transfer to induce embryo implantation. The uteri of recipient mice were collected and the number of implantation sites was counted 3 days after the embryo transfer (D10).

### Immunohistochemistry:

The paraffin-embedded uteri were sectioned (4 *μm*), deparaffinized, autoclaved (121 °*C*, 5 *min*), and incubated with 0.3% H_2_O_2_ in methanol for 30 *min* at room temperature. Polyclonal anti-PR antibodies (Sc-538, 1:100; Santa Cruz Biotechnology, Inc.; Santa Cruz, CA, USA) were applied overnight at 4 °*C* after blocking treatment. The section was incubated with biotinylated goat anti-rabbit antibody (1:800, Dako) for 1 *hr* at room temperature, followed by avidin-biotin complexation (Vector Labs; Burlingame, CA, USA) for 40 *min* at room temperature, and visualized with DAB. For the detection of ERα, deparaffinized sections were autoclaved (121 °*C*, 10 *min*) and incubated with 0.3% H_2_O_2_ in methanol for 30 *min* at room temperature. Monoclonal anti-ERα antibodies (N1575, 1:20; Dako) were used with the Histofine Mouse Stain Kit. The signals were visualized with DAB.

### High throughput sequencing:

The whole uteri were collected from pseudopregnant delayed implantation (DI) mice 24 *hr* after treatment with E2 (20 *ng* or 3 *ng*). In each group of E2 doses, the uteri collected from three individuals were mixed together, respectively. To determine mRNA expressed in these uteri, cDNA libraries for high throughput sequencing were prepared with NEB Next Ultra RNA Library Prep kit for Illumina (New England Biolabs, Ipswich, CA, USA) according to manufactures’ protocol. The obtained cDNA libraries were sequenced using a MiSeq bench-top sequencer (Illumina, San Diego, CA, USA) with 150 paired-end reads.

### RNA-Seq analysis:

The sequence data obtained from MiSeq were analyzed with CLC genomics Workbench 6.0.5 (CLC bio, Aarhus, Denmark). The obtained reads were trimmed and mapped to reference sequence set that were originated from NCBI database, which consisted of three cDNA libraries (Library ID: 1097Library ID: 1891 and 8622), and the value of average coverage was compared between 3 *ng* and 20 *ng* of E2 treated tissues.

### Quantitative real-time PCR:

The whole uteri (n=3–6) were excised from pseudopregnant DI mice 6 or 24 *hr* after treatment with E2 (20 *ng* or 3 *ng*) or corn oil (control), respectively. Total RNA was extracted with RNeasy Mini Kit (Qiagen; Hilden, Germany) and the cDNAs were synthesized from 1 *μg* total RNA with ReverTra Ace qPCR RT Kit (TOYOBO; Osaka, Japan). Real-time PCR was performed with the StepOnePlus system (Life Technologies) with SYBR Green (Life Technologies) or the LightCycler Nano system (Roche Diagnostics, Mannheim, Germany) with FastStart Essential DNA Green Master (Roche Diagnostics). The primers used in this study were as follows: Igf1, 5′-ACAGGCTATGGCTCCAGCAT-3′ and 5′-GCTCCGGAAGCAACACTCA-3′; Fgf1, 5’-GCGGGCGAAGTGTATATAAAGG-3’ and 5’-CGGTGTCCATGGCCAAGT-3’; Fgf2, 5′-CA ACCGGTACCTTGCTATGA-3′ and 5′-TCCGTG ACCGGTAAGTATTG-3′; Fgf9, 5′-CTATCCAG GGAACCAGGAAAGA-3′ and 5′-CAGGCCCA CTGCTATACTGATAAA-3′; Fgf18, 5′-GAATT CTACCTGTGTATGAACCGAAA-3′ and 5′ -TG AACACGCACTCCTTGCTAGT-3 ′; Igfbp3, 5′ -CACATCCCAAACTGTGACAA-3′ and 5′-CCA TACTTGTCCACACACCA-3′; and Rplp0 (internal control), 5′-CGACATCACAGAGCAGGC-3′ and 5′-CACCGAGGCAACAGTTGG-3′. Expression levels were normalized using the internal control (Rplp0). For the analysis of Igf1 and Igfbp3 expression, expression levels were also normalized by the values obtained in vehicle-treated mice at corresponding time points after injection. The average value for uteri treated with vehicle was considered to be 1.

### Statistical analysis:

Statistical analysis was performed by Student’s t-test and/or one-way analysis of variance (ANOVA). The p<0.05 was considered significant.

## Results

During normal pregnancy (Figure 1A), LE cell proliferation (BrdU incorporation of LE cells) was observed at D3 (Figure 1B) but not at D4 (Figure 1C). It resumed in the afternoon and/or evening of D5 in ICR mice (Figure 1D) and by D6 at the latest in C57BL/6 mice (Figures 1E, F and I). In pseudopregnant ICR mice, LE cell proliferation was not observed at the fifth day of pseudopregnancy (pD5) but resumed at pD6 (Figures 1G and H). LE cell proliferation was hindered in DI mice, where implantation was artificially inhibited by ovariectomy and continuous P4 exposure (Figure 1J). Embryos in DI mice were located close to the LE cells (apposition, figure 1K). LE cell proliferation was examined in DI mice 24 *hr* after injection with 3 *ng* or 20 *ng* E2, or 25 *μg* LIF protein; these doses were sufficient to restart the implantation processes (11, 13, 15). Treatment with the vehicle did not affect LE cell proliferation (Figure 1L). E2 (20 *ng*) induced proliferation of LE cells in DI mice (Figure 1M; the rate of proliferation was 11.13±2.35%, figure 1P). However, 3 *ng* of E2 and LIF did not show any proliferative effects on LE cells (Figure 1N-P).

**Figure 2.**
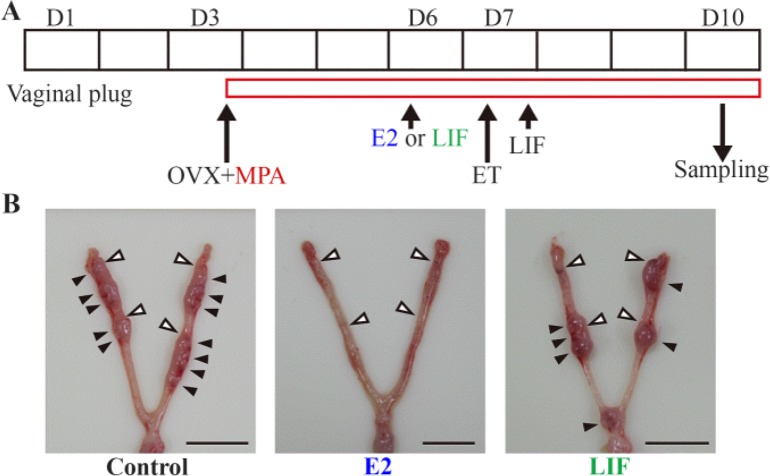
Embryo transfer to delayed implantation mice. (A) Experimental procedure for embryo transfer (B) the uteri treated with vehicle, leukemia inhibitory factor (LIF), or estrogen (E2). The solid black arrowheads point to implantation sites, and the white arrowheads point to sites where embryos were transferred. Scale bar, 10 *mm*

To confirm whether LE cell proliferation reflects uterine receptivity, an embryo transfer study was performed using ovariectomized pseudopregnant mice after treating them with E2 (20 *ng*) or LIF (25 *μg*), based on a previous report demonstrating that transferred embryos could not implant to the uteri in which high-dose E2 was treated 24 *hr* before embryo transfer (15), with several modifications (Figure 2A). When recipient mice were treated with 20 *ng* E2, 24 *hr* before embryo transfer, no implantation site was observed at D10 (n=6), consistent with a previous report (Figure 2B and Table 1) (15). Treatment with LIF (n=6) did not prevent embryo implantation, and implantation sites were also detected in the controls (no treatment, n=6) (Figure 2B and Table 1). The implantation rates (the number of implantation sites divided by the number of embryos transferred) of LIF-treated mice and control mice were 24% and 29%, respectively (Table 1).

**Table 1.** Effects of estrogen or leukemia inhibitory factor on the implantation window

**Treatment**	**Recipients, n**	**Embryos transferred, n**	**Mice with implantation sites, n (%)**	**Implantation sites, n (%)**
**Control**	6	102	6 (100)	30 (29)
**E2 (20 *ng*)**	6	100	0 (0)	0^*^ (0)
**LIF (25 *μg*)**	6	110	6 (100)	26 (24)

* One embryo was recovered from the uterus

Abbreviations: E2, estrogen; LIF, leukemia inhibitory factor

**Figure 3.**
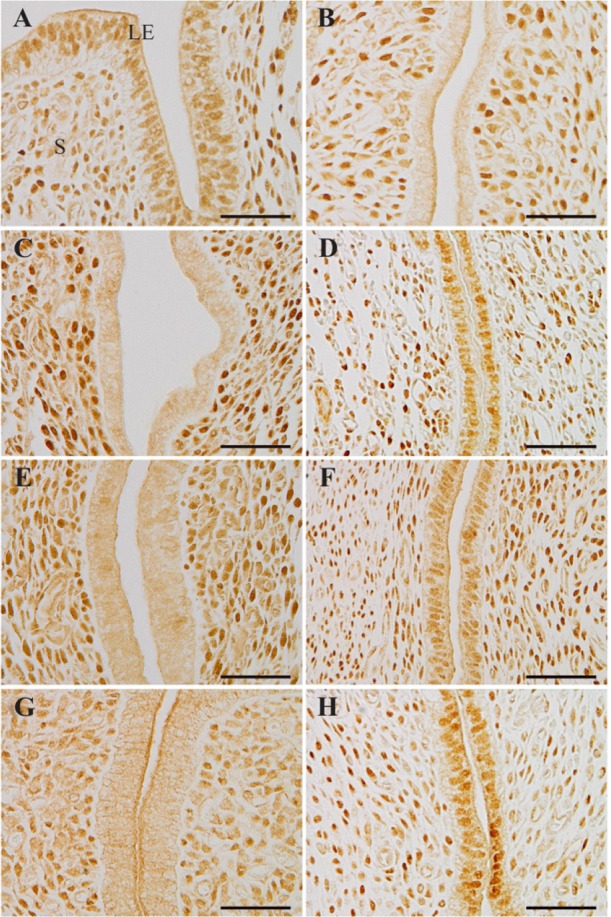
Localization of progesterone receptors. (A) The uterus of delayed implantation (DI) mouse. The DI uteri treated with 20 *ng* estrogen (E2) (B), leukemia inhibitory factor (LIF) (C, D) or 3 *ng* E2 (E, F). The uteri in ovariectomized pseudopregnant mice 24 *hr* after injection with 20 *ng* of E2 (G), or LIF (H). LE, luminal epithelium; S, stroma. Scale bar, 50 *μm*

The pattern of PR expression in the nucleus of LE cells, which was clearly associated with the uterine receptivity (16), was also analyzed in DI uteri treated with E2 or LIF. PRs were expressed in the nuclei of LE cells and the stromal cells in DI mice (Figure 3A). PRs were not observed in the nuclei of LE cells 24 *hr* after injection with 20 *ng* E2 but were still strongly expressed in the stroma (Figure 3B). LIF also reduced the expression of PRs in the nuclei of LE cells at peri-implantation sites in the same mice (Figure 3C), however, PRs were still present at the interimplantation sites (Figure 3D). Administration of low-dose E2 (3 *ng*) gave almost the same results obtained by administration of LIF (Figures 3E, F). PRs in the LE were eliminated with injection of 20 *ng* E2 in pseudopregnant ovariectomized mice (Figure 3G), but not with injection of LIF (Figure 3H).

To know which gene(s) contribute to change the endometrium to refractory at a high-dose of E2, first, the gene expression profiles in the endometrium were compared between DI mice treated with high-dose E2 (20 *ng*) and low-dose E2 (3 *ng*) at 24 *hr* by RNA-Seq deep sequencing analysis.

**Figure 4.**
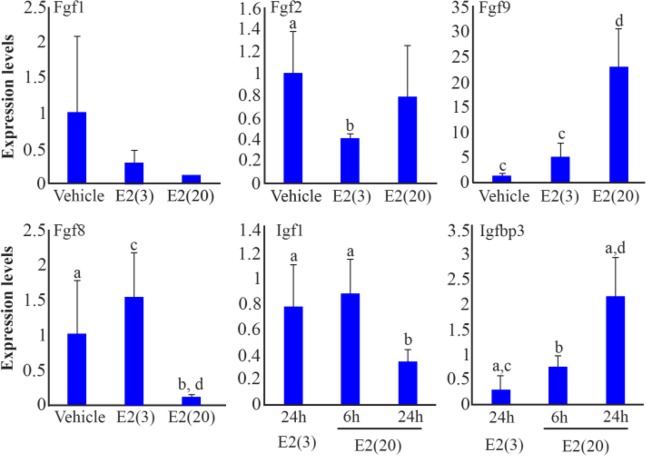
Expression of genes regulated by progesterone (P4) and estrogen (E2). Significant differences are indicated by different letters for the same molecule (average±SD, a–b: p<0.05, c–d: p<0.01)

Based on the threshold set (>4 fold change, and either of the average_coverage value was higher than 30), it was found that 19 genes were highly expressed in mice treated with high-dose E2, and 22 genes were highly expressed in mice treated with low-dose E2 (Tables 2 and 3). The expression of insulin-like growth factor binding protein 3 (Igfbp3), which was one of 19 genes that showed higher expression in mice treated with high-dose E2, was confirmed by quantitative real-time polymerase chain reaction (PCR) analysis (Figure 4). In addition, the expressions of Fgfs were investigated (especially, Fgf1, Fgf2, Fgf9, and Fgf18), which were supposed to be related to LE cell proliferation in previous studies (6), using real-time PCR analysis, since they could not be measured by RNA-Seq analysis, because they were unavailable in our reference sequences. Mice treated with 20 *ng* E2 showed lower expression levels of Fgf18 mRNA than mice treated with 3 *ng* E2 24 *hr* after administration (p<0.05 and p< 0.01, respectively, figure 4). Fgf9 (p<0.01), but not Fgf1 and Fgf2, was significantly increased in the mice treated with 20 *ng* E2 (Figure 4). The expression of Insulin-like growth factor 1 (Igf1), which was also assumed to be related to LE cell proliferation in previous studies (17) and tended to be higher in endometrium treated with low-dose E2 in our RNA-seq analysis, was also investigated using real-time PCR analysis and it was found to be significantly lower in mice at 24 *hr* after injection with high-dose E2 than in those with low-dose E2, or at 6 *hr* after injection with high-dose E2 (Figure 4).

**Table 2.** Transcripts that were highly expressed in the endometrium 24 hr after injection of high-dose E2 in RNA-Seq assay

**Reference sequence**	**E2(20 *ng*) average coverage**	**E2(3 *ng*) average coverage**	**E2(20)/E2(3)**	**Gene symbol**
**S7731261**	155.07	2.73	56.79	Olfm1
**S7727685**	56.29	1.08	51.97	8430408G22Rik
**S7730735**	108.98	5.47	19.94	8430408G22Rik
**S9039520**	228.94	11.98	19.11	Olfm1
**S7429855**	64.70	3.49	18.54	Atp6v0d2
**S9026865**	284.33	16.99	16.73	Olfm1
**S7717471**	758.43	50.38	15.05	Igfbp3
**S7482515**	915.37	61.32	14.93	Igfbp3
**S7503970**	39.30	2.90	13.54	Srd5a
**S9042223**	864.64	65.21	13.26	Igfbp3
**S9029775**	1189.64	93.29	12.75	Igfbp3
**S7493984**	43.97	5.22	8.43	Nupr1
**S7481392**	34.49	4.11	8.39	Fam213a
**S7481524**	32.70	3.91	8.35	Sema3c
**S9023759**	157.66	19.19	8.22	H6pd
**S7733293**	131.44	16.92	7.77	Ggh
**S7577403**	49.77	6.83	7.29	Transcribed locus
**S7483745**	128.41	20.55	6.25	Ndrg1
**S9019265**	171.20	27.68	6.19	Ndrg1
**S7715623**	55.21	9.20	6.00	Kank3

Twenty transcripts were selectively shown among them that fulfilled the threshold as followed; >4 fold changes, and either of the values of average coverage is upper than 30

**Table 3.** Transcripts that were highly expressed in the endometrium 24 hr after injection of low-dose E2 in RNA-Seq assay

**Reference sequence**	**E2 (20 *ng*) average coverage**	**E2 (3 *ng*) average coverage**	**E2(20)/E2(3)**	**Gene symbol**
**S9020344**	13.40	110.23	0.12	Slc2a3
**S9025835**	30.14	209.22	0.14	Fxyd4
**S18202061**	20.31	139.19	0.15	Hbb-bs
**S9033422**	14.10	96.31	0.15	Slc2a3
**S7719712**	4.64	30.18	0.15	-
**S9039123**	17.79	113.43	0.16	Hbb-bs
**S9037101**	18.56	114.00	0.16	Jam2
**S9042607**	21.54	131.89	0.16	Gstm2
**S9037119**	24.64	147.42	0.17	Gstm2
**S7734183**	39.88	235.90	0.17	Hbb-b2
**S7728782**	89.94	528.21	0.17	Hba-a1
**S9034725**	16.47	94.25	0.17	Alox12e
**S7729348**	21.63	119.56	0.18	Hba-a1
**S7732913**	6.93	37.05	0.19	-
**S18202511**	24.77	129.46	0.19	Hbb-bs
**S9027427**	17.79	91.62	0.19	Acot7
**S7720344**	20.88	106.46	0.20	Hbb-b2
**S9021753**	30.31	150.97	0.20	Alox12e
**S18202843**	32.10	159.74	0.20	Pcx
**S7716240**	19.41	95.92	0.20	Hba-a2

Twenty transcripts were selectively shown that fulfilled the threshold as followed; >4 fold changes, and either of the values of average coverage is upper than 30

-: the reference sequence was not corresponded to the gene identified previously

To confirm if the gene products are associated with transition of the endometrium to refractory *in vivo*, recombinant FGF2 and FGF9 proteins (rFGF2 and rFGF9, respectively) were administered to DI mice and LE cell proliferation was analyzed after administration. The biological activities of rFGF2 and rFGF9 were confirmed by *in vitro* cell proliferation assay using NIH-3T3 cells (data not shown) (18, 19). Proliferation of LE cells was not stimulated by 200 *μg* of rFGF2 or rFGF9 in DI mice 24 *hr* after intraperitoneal injection (n=3 each, Figure 5A-C). Next, an attempt was made to check if the treatment of recombinant IGF1 protein (Somazon, Orphan pacific) (20) *in vivo* contributed to transition of the endometrium from receptive to refractory or not. Subcutaneous injection of 100 *μg* of IGF1 in every 6 *hr* for 24 *hr* induced LE cell proliferation in DI mice (Figure 5D–G). Vehicle treatment did not induce LE cell proliferation (Figure 5H, I). The average number of proliferating cells per *mm*^2^ of the LE was 136.19 in IGF1 treated mice (n=10, Figure 5J), but 4.71 in vehicle treated mice (n=11, Figure 5J). This difference was statistically significant (p< 0.05).

## Discussion

The proliferation of LE cells at D3 and D4 during normal pregnancy in both ICR and C57BL/6 mice was consistent with previous reports (6, 21). The limited period, during which LE cell proliferation ceased (D4), coincides with the receptive phase of the uterus (1). As was expected, LE cells started to proliferate again at D5 in ICR mice, when the uterus transited to the refractory phase (Figure 1). On the other hand, re-initiation of proliferation was slightly delayed in C57BL/6 mice compared to ICR mice (by D6 in C57BL/6, figure 1F). The length of the estrus cycle (normally 4 days) varies among the strains of mice, and is relatively longer in C57BL/6 mice (22). This delayed initiation of LE cell proliferation in C57BL/6 mice may be partly explained by the physiological difference among strains. LE cell proliferation in pseudopregnant ICR mice was not observed until pD6, although it had been detected at D5 in normal pregnancy (Figure 1). Transition of serum E2 and P4 levels in pseudopregnant rodents are almost consistent with those in normal pregnancy before embryo implantation (23–25). P4 levels declined at pD6 in pseudopregnant mice (25, 26). As the anti-proliferative action of P4 on LE cells was attenuated by a reduction of serum P4 levels at pD6, cell proliferation in the LE should be automatically reinitiated at pD6 in pseudopregnant animals. LE cell proliferation at D5 in normal pregnant mice could not be explained by changes in systemic steroid hormone levels, since the transition of serum E2 and P4 shows identical patterns between normal pregnancy and pseudopregnancy until embryo implantation. After the embryo adhered to the LE in normal pregnancy, the uterine stromal cells adjacent to the embryo immediately differentiate to decidual cells (termed as deciduaslization) and synthesize E2 at the site of implantation (27). Therefore, E2 concentration at the uteri should be increased locally in pregnancy, but not in pseudopregnancy. In fact, when decidualization was artificially induced in DI mice by electric stimuli, LE cell proliferation was observed (data not shown). Thus, local E2 synthesized by decidual cells at the site of implantation induced LE cell proliferation in D5 mice and changed the endometrium to refractory.

The condition of the uteri of DI mice is usually mentioned as the neutral phase, which is defined as the period from P4 administration to the induction of embryo implantation by E2-LIF pathway in DI mice (5). When E2 or LIF was administered to DI mice, high-dose E2 (20 *ng*) stimulated LE cell proliferation after 24 *hr* (Figure 1M). LIF and low-dose E2 (3 *ng*) did not have such an effect on LE cells (Figure 1N, O). All the administered doses equally promoted embryo implantation (data not shown) (13, 15). In addition, an embryo transfer study clearly showed that the uterus became refractory with administration of high-dose E2 (20 *ng*) but not of LIF (Figure 2). It was postulated that the nuclear localization of PRs in the LE is also associated with the phase of the uterus (16). PRs are strongly expressed in the LE before embryo implantation (D3), but are downregulated after adhesion of the embryo to the LE (D5) (16). As expected, PRs were not observed in the nuclei of LE cells along the uterine horn 24 *hr* after injection of 20 *ng* of E2 in DI mice (Figure 3A, B). LIF and low-dose E2 (3 *ng*) also reduced the expression of PRs in the nuclei of LE cells at peri-implantation sites (Figures 3C, E); however, PRs were still present at the interimplantation sites (Figure 3D, F). Partial disappearance of PRs at peri-implantation sites could happen because of the higher E2 effect at the site of implantation, where E2 is locally synthesized de novo as discussed above (27). Considered together, these results demonstrated that high-dose E2 potentiates a change of the endometrial receptivity to the refractory phase via a pathway different from the LIF pathway. A previous study indicated that E2 determined the duration of the receptive phase in a dose-dependent manner, affecting the expression of implantation-related genes, including Lif and prostaglandin-endoperoxide synthase 2 (Ptgs2) (15). The lower dose (3 *ng*) could sustain the receptive phase for a longer period than a higher dose (>10 *ng*) (15). In other words, a low dose of E2 could not change the phase of the uterus; only a higher dose will make the receptive uterus refractory. These facts suggested that the uterus does not automatically become refractory to embryos as previously thought, and that this process is precisely controlled by E2.

Subepithelial estrogen receptor α(ERα), but not ERα in the LE, plays a critical role in the proliferation of LE cells, suggesting that the mitogenic action of E2 on the LE is mediated by stromal derived paracrine molecules (28). The candidates for such mitogenic mediators are the members of FGF family, such as FGF1, FGF2, FGF9 and FGF18 (6). Decreased expressions of these transcripts by P4-HAND2 signaling correlate with termination of LE cell proliferation in the receptive phase of the endometrium (6). Our results showed that high-dose E2 treatment increased only Fgf9 expression in these members (Figure 4), and even rFGF9 injection into DI mice as well as rFGF2 injection could not induce LE cell proliferation (Figures 5A, B). These results suggested that, at least, FGF2 and FGF9 were not involved in E2-dependent reinitiation of LE cell proliferation in pregnant mice; additionally, FGF1 and FGF18 did not seem to be involved, because these transcripts were not upregulated by E2 treatment (Figure 4). The contribution of the IGF1 signaling to change the endometrium to refractory was also studied. IGF1 has been investigated as a paracrine mediator of the mitogenic effect of E2 on uterine LE cells (17). E2 treatment activates the IGF1 signaling through the phosphorylation of IGF1 receptor in the LE cells of adult mice (29). Several reports demonstrate that E2 increases the expression of uterine Igf1 mRNA in ovariectomized rodents (30, 31); however, in our experiment, Igf1 expression was not upregulated in the endometrium of DI mice following E2 treatment (Figure 4). On the other hand, it was confirmed that injection of IGF1 protein stimulated LE cell proliferation in DI mice. Although the amount of IGF1 injected to mice here (total 400 *μg* per mice) seemed to be at supraphysiologic levels, the same or higher IGF1 administration never caused destructive effects on homeostasis of rodents in the previous reports (32, 33); then it was concluded that the present of IGF1, solely on the uterus, could be evaluated properly. The effect of IGF1 on proliferation was less extensive than the response to high-dose E2 (comparison of Figure 1M to Figures 5D, E), but it was consistent with the previous reports (34). This implied that LE cell proliferation during the peri-implantation period was induced by E2 through activation of the IGF1 signaling, without upregulation of IGF1 ligand expression. Interestingly, our RNA-Seq and real-time PCR analysis showed that the expression of Igfbp3 was higher in DI mice treated with high-dose E2 than in mice treated with low-dose E2 (Figure 4 and Table 2). IGFBP3 forms a ternary complex with IGF1 and the acid-labile subunit (ALS) to amplify the half-life of IGF1 in circulation and modulate actions of IGF1 (35). The expression of Igfbp3 is observed in the endometrial epithelium as well as in the subepithelial stroma during early pregnancy (36), but the significance of locally synthesized IGFBP3 in murine endometrium remains poorly understood. There is evidence that IGFBP3 in the pericellular environment enhances the presentation of IGF1 to its receptor and potentiates IGF1 actions *in vitro* (37, 38). Thus, it was hypothesized that upregulated IGFBP3 in the endometrium followed by E2 stimuli would contribute to LE cell proliferation through recruitment of systemic and/or local IGF1 to the IGF1 receptor on the cellular membrane of LE. Taken together, the effect of E2 for changing the endometrium to refractory state is mediated by subepithelial stromal ERα that induces the expression of Igfbp3, which enhances the IGF1 signaling in the LE.

## Conclusion

The murine uterus becomes receptive to embryos by the sole action of P4, mediated by HAND2. Nidatory E2 immediately stimulates the uterine glands to secrete LIF, which promotes transition from embryo apposition to adhesion to the LE through an undefined downstream pathway. Following embryo adhesion, uterine stromal cells immediately undergo differentiation, known as decidualization, and begin synthesizing E2 at the site of implantation. Locally produced E2 makes the uterus refractory by activating the IGF1 signaling on the LE with IGFBP3 action. In this study, the regulatory mechanism of transition of the endometrium from receptive to refractory was elucidated, which means the closure of “the implantation window”. Since the failure of appropriate regulation of the window is one of the causes of miscarriage during early pregnancy in humans, our findings will provide beneficial knowledge for fertility treatment for humans.
